# Correction to: Psychosocial factors associated with quality of life in cancer survivors: umbrella review

**DOI:** 10.1007/s00432-024-05929-6

**Published:** 2024-09-02

**Authors:** Viktorya Voskanyan, Chiara Marzorati, Diana Sala, Roberto Grasso, Ricardo Pietrobon, Iris van der Heide, Merel Engelaar, Nanne Bos, Augusto Caraceni, Norbert Couspel, Montse Ferrer, Mogens Groenvold, Stein Kaasa, Claudio Lombardo, Aude Sirven, Hugo Vachon, Galina Velikova, Cinzia Brunelli, Giovanni Apolone, Gabriella Pravettoni

**Affiliations:** 1 Applied Research Division for Cognitive and Psychological Science, IEO, European Institute of Oncology IRCCS, Milan, Italy; 2 Department of Oncology and Hemato-Oncology, University of Milan, Milan, Italy; 3 SporeData, Inc., Durham, NC USA; 4 Nivel, Netherlands Institute for Health Services Research, Utrecht, Netherlands; 5 Dipartimento Di Scienze Cliniche E Di Comunità, Università Degli Studi Di Milano, Milan, Italy; 6 European Cancer Organisation, Brussels, Belgium; 7 Health Services Research Group, Hospital del Mar Research Institute, Barcelona, Spain; 8 Department of Public Health, Bispebjerg Hospital and University of Copenhagen, Copenhagen, Denmark; 9 Department of Oncology, Oslo University Hospital, Oslo, Norway; 10 OECI-EEIG Organisation of European Cancer Institutes—European Economic Interest Grouping, Brussels, Belgium; 11 UNICANCER, Paris, France; 12 EORTC, Brussels, Belgium; 13 Leeds Institute of Medical Research at St James’s, University of Leeds, Leeds, UK; 14 Leeds Cancer Centre, Leeds Teaching Hospitals NHS Trust, Leeds, UK; 15 Scientific Directorate, Fondazione Istituto Di Ricovero E Cura a Carattere Scientifico, Fondazione IRCCS Istituto Nazionale Dei Tumori, Milan, Italy

**Correction to: Journal of Cancer Research and Clinical Oncology (2024) 150:249** 10.1007/s00432-024-05749-8

In this article, the author’s name Iris van der Heide was incorrectly written as Iris van der Heijden.

In addition, the affiliation details for Cinzia Brunelli were incorrectly given as 'Dipartimento Di Scienze Cliniche E Di Comunità, Università Degli Studi Di Milano, Milan, Italy' but should have been 'Scientific Directorate, Fondazione Istituto Di Ricovero E Cura a Carattere Scientifico, Fondazione IRCCS Istituto Nazionale Dei Tumori, Milan, Italy '.

The original article has been corrected.

